# Supplementary material for: Long-Term Pioglitazone Treatment Has No Significant Impact on Microglial Activation and Tau Pathology in P301S Mice
Source: Int J Mol Sci. 2023 Jun 14;24(12):10106. doi: 10.3390/ijms241210106 (PMC10298936; doi:10.3390/ijms241210106)
Supplement: Supplementary file 1 [file ijms-24-10106-s001.zip › ijms-2381530-supplementary.pdf]

Supplemental Figures

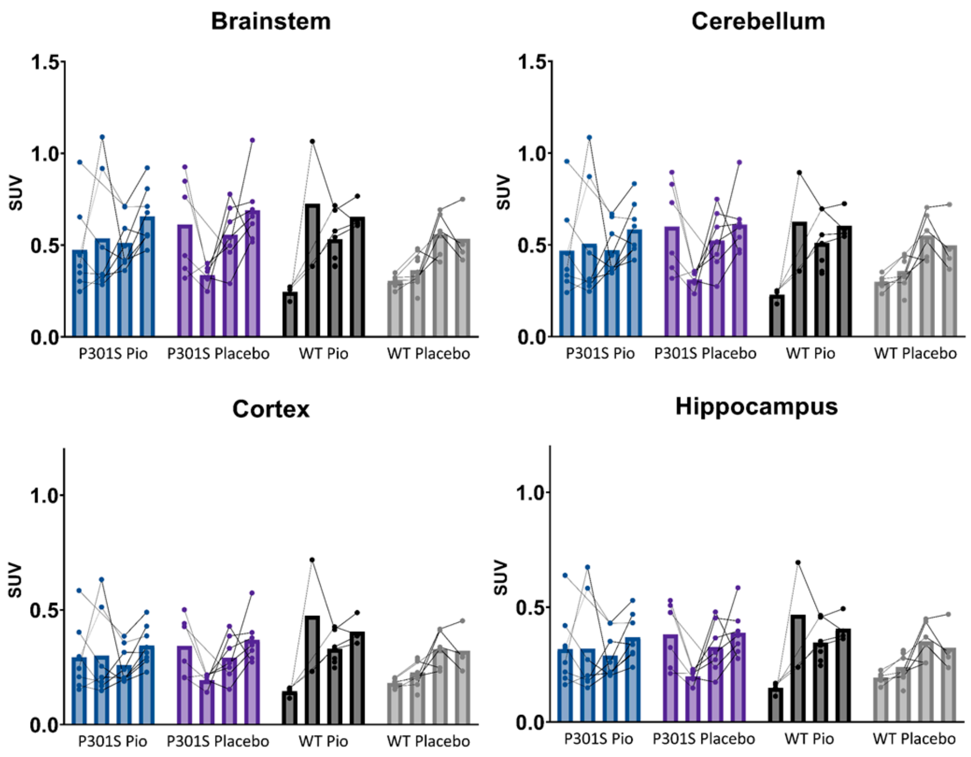

Figure S1. Individual time courses of SUV-scaled TSPO-PET signals of P301S mice treated with pioglitazone (P301S Pio) or placebo chow (P301S Placebo) and the respective wild-type (WT) control groups over time in brainstem, cerebellum, cortex, and hippocampus.

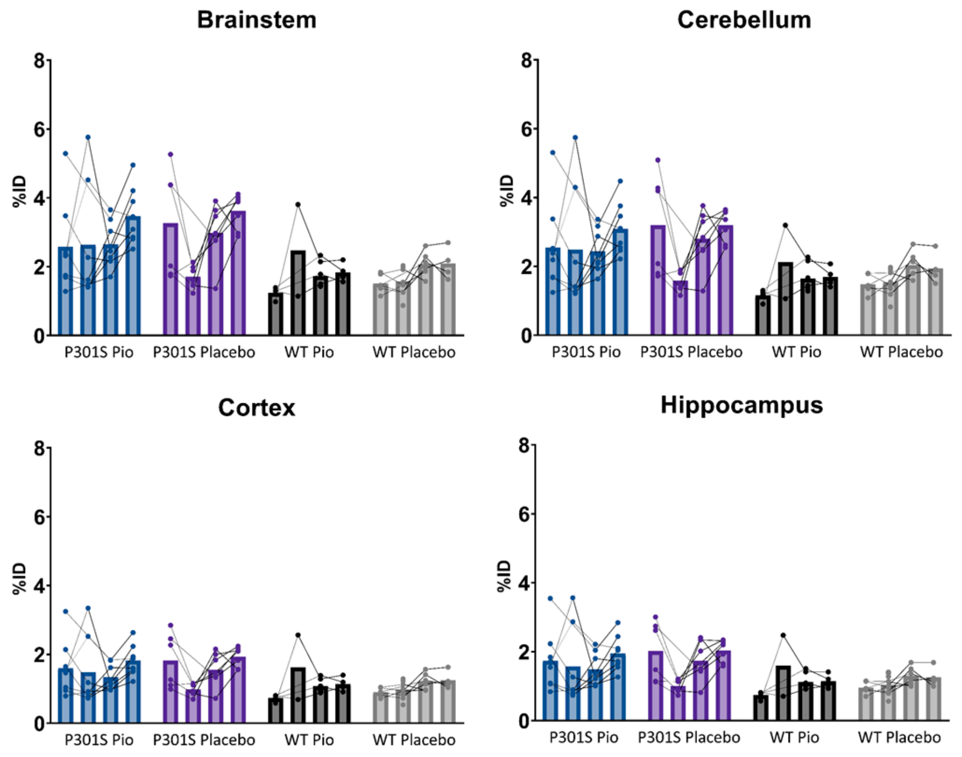

Figure S2. Individual time courses of %ID-scaled TSPO-PET signals of P301S mice treated with pioglitazone (P301S Pio) or placebo chow (P301S Placebo)

and the respective wild-type (WT) control groups over time in brainstem, cerebellum, cortex, and hippocampus.

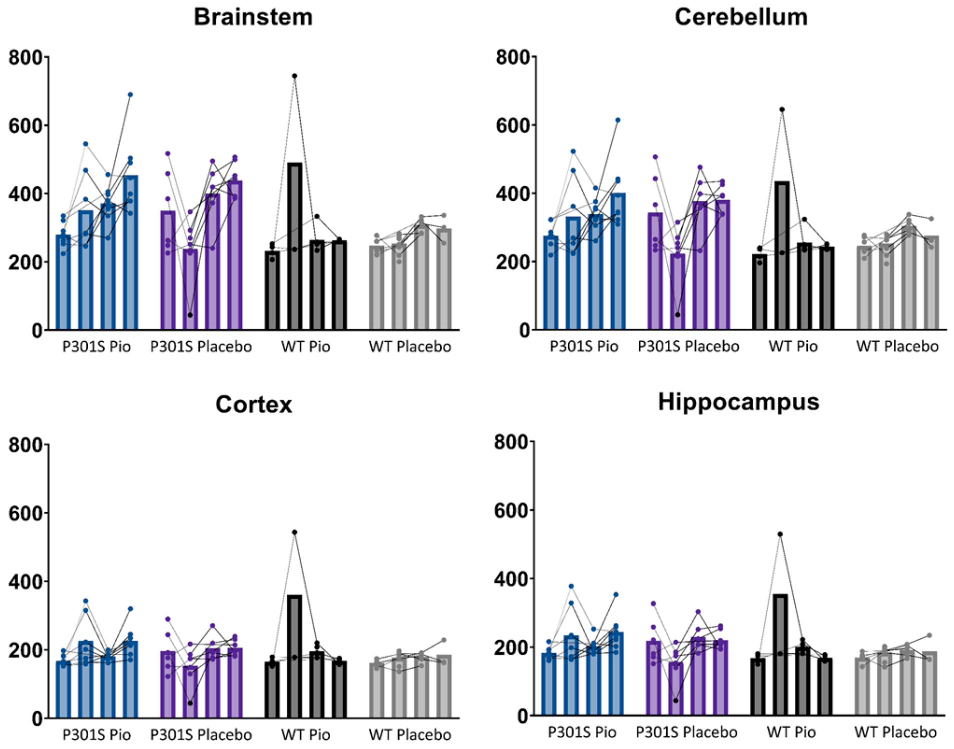

Figure S3. Individual time courses of myocardium-scaled TSPO-PET signals of P301S mice treated with pioglitazone (P301S Pio) or placebo chow (P301S Placebo) and the respective wild-type (WT) control groups over time in brainstem, cerebellum, cortex, and hippocampus.
